# Investigating the Quality of Life for Cancer Patients and Estimating the Cost of Immunotherapy in Selected Cases

**DOI:** 10.7759/cureus.32390

**Published:** 2022-12-10

**Authors:** Georgios F Fragkiadakis, Maria Spiliotopoulou

**Affiliations:** 1 Social Sciences School, Hellenic Open University, Chania, GRC; 2 Oncology Department, Day Care Unit/Nursing, General University Hospital of Patras, Patra, GRC

**Keywords:** cost effective therapy, health care outcomes, cancer patients, quality of life (qol), cancer immunotherapy

## Abstract

The aim of this study is to assess the quality of life of patients receiving immune checkpoint inhibitors (ICI) by evaluating their physical and psychological well-being as well as their social and spiritual functioning. The 36-item Short Form Health Survey (SF-36) and quality of life core 30 (QLQ-C30) questionnaires were used to measure the quality of life of people receiving checkpoint inhibitors. An attempt was also made to make a rough estimate of the cost of checkpoint inhibitors in selected cases. The present study was conducted at the Oncology Day Hospital of the General University Hospital of Patras and the sample consisted of 100 subjects. The results of the two questionnaires show that the subjective evaluation of the patient's quality of life is satisfactory and functional since most of the respondents evaluate their quality of life as good to very good. Regarding the duration of immunotherapy, their health status seems to have improved, as 49% of respondents report having no pain, while the QLQ-C30 shows that 93% of patients have no problems with personal hygiene. Important determinants related to the limitation of work and daily activities were influenced by patients' age and marital status. Finally, age, monthly income, and education level seem to exert a general influence on a person's physical condition.

## Introduction

An important issue of concern to the scientific community is the evaluation of the quality of life of cancer patients since many deaths worldwide are due to cancer. The innovation of immunotherapy aims to cure cancer, improve the quality of life of cancer patients, and increase survival rates. Immunotherapy aims to treat tumors such as lung cancer, kidney cancer, melanoma, and head and neck cancer by activating each person's immune system to its advantage. Until the end of the last decade, cancer treatment options included surgery, radiotherapy, and chemotherapy [1,2]. In 2000, immunotherapy emerged as a treatment for cancer that activates the body's immune system by using it to destroy cancer cells. The innovation of immunotherapy is divided into active, passive, and inductive immunotherapy [3]. Active immunotherapy was initially done by vaccination with the aim of inducing cancer-like reactions. Active immunotherapies include the unique Sipuleucel-T vaccine, which has been administered for the treatment of metastatic hormone-refractory prostate cancer since 2010 [4]. This is followed by passive immunotherapy with the administration of monoclonal antibodies that inhibit the growth of cancer foci. Finally, inductive immunotherapy is a type of transplantation of ex vivo-modified immune cells that act as cancer-specific receptors [5].

The diversity of immunotherapy in the treatment of various cancers has shown extremely promising results, even in combination with previous therapeutic methods, improving the clinical outcomes and quality of life of cancer patients and significantly prolonging their survival [6]. Consequently, immunotherapy is actively involved in the treatment of cancer. The discovery of immunoregulatory receptors called "immune checkpoint inhibitors" (ICI) in cancer cells and defense cells in the lymphatic system was the beginning of the development of drugs that inhibit the function of the receptors and thus alter the immune mechanism that acts as a defense for the patient [7]. The return to normality, long-term survival of patients, low side effects, and unique nature of treatment make immunotherapy a means of first- and second-line treatment of metastatic non-small cell lung cancer (NSCLC), as well as a means of care after chemotherapy and radiotherapy [8].

Background

The efficacy of immunotherapeutics and their impact on patients' quality of life are reflected in phase 3 - KeyNote-024 study, which evaluated the efficacy of pembrolizumab compared with standard chemotherapy for patients with advanced NSCLC. The study was conducted on 305 patients who received a fixed dose of 200 mg of pembrolizumab for 35 cycles. At the end of the study, longer survival was observed, averaging 10.3 months with pembrolizumab administration compared with six months with chemotherapy administration. The percent survival for pembrolizumab in NSCLC was 80.2% versus 72.4% with chemotherapy. There was also no progression of malignancy and fewer side effects compared with chemotherapy. The study enrolled patients with a mean age of 65 years, 61% of whom were male [9].

In addition, the Checkmate-017 phase 3 study is evaluating the efficacy of nivolumab for patients with advanced squamous-cell NSCLC. The study enrolled 272 patients over 18 years of age who received nivolumab at a dose of 3 mg per kilogram of body weight for two weeks, compared to chemotherapy with docetaxel 75 mg. The results of the study showed an overall survival of 9.2 months with nivolumab versus six months with chemotherapy. At one year, the overall survival rate was 42% for nivolumab and 24% for docetaxel. In conclusion, patients with advanced lung cancer had a higher progression-free survival rate thanks to nivolumab [10].

The Impower-130 study was conducted on 724 patients over 18 years of age with cytologically confirmed stage IV NSCLC who received the monoclonal antibody atezolizumab PDL-1 every three weeks at a dose of 1200 mg. The results of the study showed a significant clinical improvement in life expectancy as well as significant progression-free survival. The survival months with atezolizumab administration were 18.6 months, compared to 14 months with chemotherapy [11].

Finally, regarding the drug durvalumab, in the Pacific study, 473 patients with NSCLC, stage 3, were given 10 mg of durvalumab per kilogram of body weight every two weeks for up to one year, and 236 were given a placebo. The study conclusions were a longer survival of up to 16.8 months without disease progression in those given durvalumab, rather than the placebo of up to 5.6 months survival [12].

## Materials and methods

Two quality-of-life assessment questionnaires, the quality-of-life core 30 (QLQ-C30) and the 36-item Short Form Health Survey (SF-36), were completed by patients actively receiving immunotherapeutic drugs to collect data. The QLQ includes 30 questions developed by the European Agency for Research and Treatment of Cancer to assess the health-related quality of life of cancer patients. The questionnaire is a valid and reliable instrument for breast cancer patients and end-stage patients [13,14]. The scales of the questionnaire in this paper consist of five measures of the individual's functioning (emotional, physical, cognitive, social, and role) and nine symptom scales to control vomiting, nausea, pain, sleep disturbance, fatigue, shortness of breath, constipation, and diarrhea, as well as a health and quality of life dimension.

The second questionnaire, the SF-36, consists of 36 questions on eight dimensions and is a general instrument for self-assessment of quality of life, with the time span covering the periods before, during, and after completion of treatment [15,16]. The eight dimensions relate to physical functioning, physical role, physical pain, general health, vitality, social functioning, emotional role, and mental health. All of the previous scales relate to both physical and mental health.

The sample (Table 1) consisted of 100 patients, 85 of whom had lung cancer, and the rest had another type of cancer-55 had NSLCL and 30 had SCLC. The sample consisted of 63% men and 37% women, with a mean age of 60 years. Questionnaires were distributed to approximately 150 individuals, 100 of whom responded completely. The researcher helped these patients complete the questionnaires while they were in the hospital. Patients were over 18 years old, had achieved a durable response to an ICI, and had been followed up for at least 12 months after starting ICI treatment without disease progression after receiving at least one dose of ipilimumab, nivolumab, pembrolizumab, or a mixture. Most patients were between 50 and 70 years old (82%), and they received their therapy according to the health protocol of the General Hospital of the University of Patras. The study was approved by a research ethics committee, and no sensitive information was used. Final approval was granted by the Board of Directors of the General Hospital of the University of Patras on February 4, 2021.

**Table 1:** Demographic statistics

		ν	%
Gender	Male	63	63%
	Female	37	37%
Age	19–29	4	4%
	30–39	0	0%
	40–49	14	14%
	50–59	21	21%
	60–69	40	40%
	70+	21	21%
Marital status	Married	71	71%
	Single	15	15%
	Widowed	7	7%
	Divorced	7	7%
Educational level	Primary school	25	25%
	Secondary school	50	50%
	Higher education/university	25	25%
Employment status	Employed	20	20%
	Unemployed	21	21%
	Retired	59	59%

## Results

In the research process derived from the QLQ-C30, 73% of patients answered (no missing answers) that they experienced little or no discomfort during strenuous work. In the case of patients having to take a long or short walk, 79% and 70%, respectively, reported that they experienced no discomfort. In addition, immunotherapy does not negatively affect patients' behavior during treatment, with 81% of patients responding that they experience little or no discomfort when they do not have to stay in a chair or in bed. Ninety-three percent of patients answer that they perform their own personal hygiene. A positive impression of ICI is also that they are not restricted in their work or daily activities, as 73% answer that they have not been restricted. In the area of hobby activities, 81% of patients have not been restricted; thus, their daily life is not affected by the treatment they receive. According to the results of the QLQ-C30, the presence of shortness of breath is absent in 81% of cases, while only 1% answered that they wheeze a lot. When asked about pain, 87% said they had none to very little. Thus, immunotherapy treatment does not cause pain sensations that interfere with patients' daily lives in all dimensions. Overall, the results show that 84% of patients experienced no or very little loss of appetite, as there were no cases of constipation or diarrhea. A high percentage of patients, 82% of the sample, do not feel tired, and 88% have no pain. As for the depressing points in the daily lives of the patients, 83% of the respondents answered that they do not feel depressed or distressed. According to the study, checkpoint

inhibitors do not prevent patients from responding to their family life, as 88% of the respondents stated that they did not experience any difficulties, and a high percentage of 48% noted that they were happy in the patients' lives (Table 2).

**Table 2:** Summary results of QLQ-C30

	Not at All	A Little	Quite a Bit	Very Much
1. Do you have any trouble doing strenuous activities, like carrying a heavy shopping bag or a suitcase?	30%	43%	22%	5%
2. Do you have any trouble taking a long walk?	34%	45%	17%	4%
3. Do you have any trouble taking a short walk outside of the house?	70%	25%	5%	0%
4. Do you need to stay in bed or a chair during the day?	37%	44%	14%	5%
5. Do you need help with eating, dressing, washing yourself, or using the toilet?	93%	4%	0%	3%
During the past week				
6. Were you limited in doing either your work or other daily activities?	51%	30%	18%	1%
7. Were you limited in pursuing your hobbies or other leisure time activities?	57%	24%	19%	0%
8. Were you short of breath?	41%	40%	18%	1%
9. Have you had pain?	70%	17%	10%	3%
10. Did you need to rest?	21%	50%	29%	0%
11. Have you had trouble sleeping?	55%	23%	16%	6%
12. Have you felt weak?	51%	33%	11%	5%
13. Have you lacked appetite?	76%	15%	7%	2%
14. Have you felt nauseated?	85%	8%	5%	2%
15. Have you vomited?	93%	3%	4%	0%
16. Have you been constipated?	61%	20%	16%	3%
17. Have you had diarrhea?	80%	16%	3%	1%
18. Were you tired?	47%	35%	15%	3%
19. Did pain interfere with your daily activities?	66%	22%	8%	4%
20. Have you had difficulty in concentrating on things, like reading a newspaper or watching television?	80%	15%	3%	2%
21. Did you feel tense?	61%	25%	13%	1%
22. Did you worry?	47%	29%	20%	4%
23. Did you feel irritable?	58%	25%	9%	8%
24. Did you feel depressed?	70%	16%	10%	4%
25. Have you had difficulty remembering things?	65%	20%	13%	2%
26. Has your physical condition or medical treatment interfered with your family life?	72%	16%	8%	4%
27. Has your physical condition or medical treatment interfered with your social activities?	62%	26%	7%	5%
28. Has your physical condition or medical treatment caused you financial difficulties?	45%	28%	24%	3%

**Figure 1:**
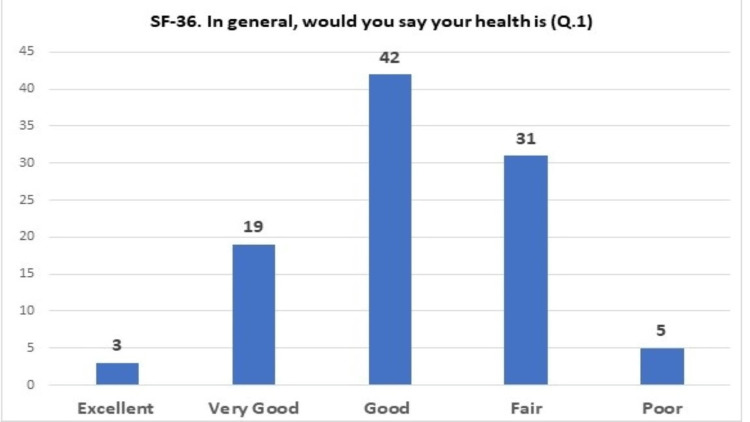
Patients' view of their level of health (SF-36)

Regarding the SF-36 questionnaire, 61% of patients feel that their overall health is good to very good (Figure 1). However, a small percentage, reaching 3%, described it as excellent; thus, patients perceive the severity of the disease and their condition. Compared with a year ago, 33% of the total sample reported that their health status remained about the same, 47% described it as somewhat better to very much better, and 18% rated the outcome of treatment in favor of their health negatively. Table 3 breaks down the results by gender, education level, and age distribution in question 2 of the SF-36 questionnaire on patients' health status compared with a year ago. Fifty percent of both men and women reported that their health status had improved compared to one year ago. Regarding the level of education, it can be seen that those who have completed primary and secondary education rate their health better than those who have completed higher education. As for the age distribution, older people consider their health better than a year ago.

**Table 3:** Evaluation of health level compared to one year ago by gender, educational level, and age (SF-36, Q.2)

SF-36. Q2. Compared to one year ago, how would you rate your health in general now?				
		Much better or/and better now than one-year ago	About the same	Somewhat worse now or/and much worse than one-year ago
Gender	Male	51% (32)	33% (21)	16% (10)
	Female	46% (17)	32% (12)	22% (8)
Educational level	Primary school	52% (13)	40% (10)	8% (2)
	Secondary school	52% (26)	32% (16)	16% (8)
	Higher education	40% (10)	28% (7)	32% (8)
Age group	19–29	50% (2)	0% (0)	50% (2)
	30–39	0% (0)	0% (0)	0% (0)
	40–49	57% (8)	7% (1)	36% (5)
	50–59	52% (11)	38% (8)	10% (2)
	60–69	40% (16)	45% (18)	15% (6)
	70+	57% (12)	29% (6)	14% (3)

However, 42% of respondents indicated that they are not as well off as they would like to be, but without giving a reason. Compared with the pain-related question of the QLQ-C30 questionnaire and the SF-36, a similarity in terms of responses was observed, as 63% of respondents in the SF-36 questionnaire considered that immunotherapy did not cause episodes of physical pain. At the psychological level, 39% of patients undergoing immunotherapy do not seem to experience psychological impairment. This is likely due to the treatment process, which does not require patients to stay in the hospital for long periods of time, and the reduced side effects. When asked at 9H about their feelings of happiness, 48% of patients give positive answers, and only 8% do not feel happy at all. It is worth noting that happiness is a multifaceted state influenced by various aspects of daily life. When asked about feeling tired, 39% of respondents said that they did not feel tired at all or felt somewhat tired.

Quality of life assessment and cost estimation in selected cases

The present study focused on selected cases to capture specifics related to the quality of life during immune checkpoint inhibitor therapy and the resulting costs. Case-related costs and treatment duration are reported to present an estimate of the cost of immunotherapy with checkpoint inhibitors in the country's healthcare system among the different drugs administered. The cost of administering immunotherapeutic drugs was calculated according to the pricing system of the Ministry of Health, as is the case for all medical interventions in public structures. Only the direct costs of the drugs used to treat patients during their stay in the oncology clinic are included in the survey (Table 4).

**Table 4:** Cost of immunotherapeutic drugs of selected cases

	Days of treatment	Time interval treatments	Cost
Case Α - Nivolumab	1.523	109	242.146,77 €
Case B - Nivolumab	1.037	74	164.393,22 €
Case C - Atezolizumab	472	23	81.272,00 €
Case D - Pembrolizumab	761	37	173.697,00 €
Case Ε - Avelumab	904	65	289.361,00 €
Min	472	23	81.272,00 €
Max	1.523	109	289.361,00 €
Average	939	62	190.174,00 €

Case A

A 53-year-old female patient with metastatic melanoma began treatment with nivolumab after receiving chemotherapy for the disease. Nivolumab treatment initiation was reported as May 8, 2017, with a dosing of 177 mg/21 days for the first four treatments and 240 mg/14 days continuously thereafter. The patient will receive the exact dosage until July 8, 2021. The patient has not experienced any toxicity from the drug; urea and creatinine levels remain at stable levels. According to CT, disease progression is stable and has not worsened. In terms of quality of life, the patient has no physical pain, is not limited in her daily tasks, and works normally. In addition, she is not limited in her social obligations, and her energy has not decreased.

Case B

A female patient, 48 years old, has non-small cell lung cancer. After six cycles of chemotherapy, immunotherapy with nivolumab 240 mg/14 days was started on September 4, 2018. The patient has not experienced any drug toxicity. The disease seems to have responded to treatment, as it has not shown any worsening. The patient's blood tests were within normal ranges during the treatments. The patient will receive nivolumab until July 6, 2021, with no impact on his quality of life. There are no signs of physical fatigue or pain, and no psychological effects. In addition, the patient is working normally and fulfilling her daily obligations.

Case C

A 68-year-old female patient with small-cell lung cancer has started immunotherapy treatment for the disease after completing chemotherapeutic treatment of the tumor. The patient has been receiving atezolizumab 1200 mg/21 days since March 9, 2020. Radiographs have shown no exacerbation of the disease. The patient has not shown any skin, endocrinologic, or hematologic instability that would require discontinuation of administration. The patient's quality of life has not been affected, as she has little pain, can do things of daily living, and her health appears to be improving. The last administration of atezolizumab to this patient occurred on June 23, 2021.

Case D

A 48-year-old male patient with metastatic renal cancer started immunotherapy after chemotherapy. Pembrolizumab 200 mg was administered every 21 days. The first administration of pembrolizumab took place on June 3, 2019. In his general health, the patient has not shown signs that require a doctor's order to stop the drug, such as toxicity or side effects that require medical monitoring. In addition, the patient's quality of life is in a state that is not limited to coping with physical exertion. He also shows no severe signs of pain or mental instability. The last administration of pembrolizumab was on May 26, 2021, as controls show that the disease is responding positively to the immunotherapeutic treatment of cancer.

Case E

A 53-year-old male patient with bladder cancer started treatment with avelumab 1,090 mg/14 days after four cycles of chemotherapy. The first administration of the drug took place on January 11, 2019. During treatment, the patient did not experience dermatological side effects, endocrinological side effects, or side effects in other parts of the body. The general picture of the patients in the control group showed no signs of toxicity or deterioration. The patient continued taking the drug until July 2, 2021. The patient's quality of life is without physical or mental limitations. He also does not feel any pain, and his personal health assessment is much better compared to one year ago.

## Discussion

From the results of this study, some critical conclusions emerge regarding both the socioeconomic characteristics of the subjects and their mental health in relation to the choice of checkpoint inhibitors as a method of cancer treatment. A statistically significant relationship was found between the age, marital status, and income of the patients in relation to the score they assigned to the overall health status assessment. The study showed a positive correlation (Table 5) with patients' age (p=0.001 < 0.05) and marital status (p=0.019 < 0.05), as reported in related studies [17]. Specifically, patients who answered question 29 of the QLQ-30 and were older rated their health status higher (5.9 out of 7) than patients who were younger, and they rated it 4.9 on average. Furthermore, higher-income patients rated their health status lower than patients with lower incomes. Regarding the family situation, single and divorced people rated their health status as "very good" or "excellent," while married people rated it as "average" or "good."

**Table 5:** Demographic characteristics that influence or relate to the patient's overall health assessment QLQ-C30 (Q.29).

Demographic characteristics	How would you rate your overall health during the past week?
Age	p = 0.001 < 0.05
Gender	p = 0.448 > 0.05
Marital status	p = 0.019 < 0.05
Residence area	p = 0.207 > 0.05
Educational level	p = 0.242 > 0.05
Employment status	p = 0.122 > 0.05
Monthly income	p = 0.043 < 0.05

According to the SF-36, not only do age, marital status, and income level affect patients' quality of life, but the level of education also seems to have an impact on an individual's family life and health status. In particular, patients with a university degree seem to rate their health quality lower than patients who do not have a university degree. However, respondents' health assessment based on marital status and monthly income is a parameter that assesses patients' quality of life in the same way as QLQ-30 scores. In addition, place of residence and monthly income influence how patients assess their health (Table 6), according to the SF-36 ΗQOL instrument. In contrast, when psychology is reduced, all demographic characteristics influence the response except gender and place of residence.

**Table 6:** Demographic characteristics that influence or relate to the patient's overall health assessment SF-36 (Q.1).

Demographic characteristics	In general, would you say your health is
Age	p = 0.000 < 0.05
Gender	p = 0.656 > 0.05
Marital status	p = 0.446 > 0.05
Residence area	p = 0.021 < 0.05
Educational level	p = 0.022 < 0.05
Employment status	p = 0.761 > 0.05
Monthly income	p = 0.218 > 0.05

In our study, we tried to evaluate the quality of life of cancer patients who underwent the method of ICI, as other researchers have done in the past [18-21]. Similar studies, also, have found a statistically significant association between socioeconomic and demographic characteristics and quality of life in cancer patients, such as marital status, age, and gender [22-25]. The present study highlights the severity of the disease but also the benefits of checkpoint inhibitor therapy on quality of life during and after treatment and is consistent with related studies [26-28]. However, a systematic study is needed at the national level in Greece to collect the necessary critical information on the course of the disease in cases monitored by hospitals in the country. A limitation of this work is the lack of access to the medical records of individual patients to collect additional information on the evolution and course of treatment.

## Conclusions

The use of ICI is an innovative way to treat cancer. For this reason, it is necessary to conduct training activities for nurses and medical staff on the different types of immunotherapy. Recognizing the needs of each patient and assessing their quality of life during treatment are key elements of effective and quality health care. Treatment and care are not limited to the hospital but also extend to outpatient care and information for oncology patients, which can provide valuable information about disease progression and treatment effectiveness.

Finally, it is important to evaluate new therapeutic procedures in terms of patient survival and the resulting quality of life. The development of interpersonal relationships between patients and the therapeutic team is also considered important, and patient participation in decision-making is a motivation to recognize the problems they face.
